# The Protective Effects of Cobra Venom from *Naja naja atra* on Acute and Chronic Nephropathy

**DOI:** 10.1155/2013/478049

**Published:** 2013-07-25

**Authors:** Shu-Zhi Wang, He He, Rong Han, Jia-Li Zhu, Jian-Qun Kou, Xiao-Lan Ding, Zheng-Hong Qin

**Affiliations:** Department of Pharmacology and Laboratory of Aging and Nervous Diseases, Soochow University School of Pharmaceutical Science, 199 Ren Ai Road, Suzhou 215123, China

## Abstract

This study investigated the effects of *Naja naja atra* venom (NNAV) on acute and chronic nephropathy in rats. Rats received 6 mg/kg adriamycin (ADR) once to evoke the chronic nephropathy or 8 ml/kg 50% v/v glycerol to produce acute renal failure (ARF). The NNAV was given orally once a day starting five days prior to ADR or glycerol injection and continued to the end of experiments. The animals were placed in metabolic cages for 24 h for urine collection for urinary protein determination. The kidney function-related biochemical changes and index of oxidative stress were determined with automatic biochemistry analyzer or colorimetric enzyme assay kits. The pathomorphological changes were observed using light and transmission electron microcopies. The levels of inflammatory cytokines and NF-**κ**B activation were determined using ELISA kits, Western blot analysis, or immunofluorescence. The results showed that NNAV relieved ADR-induced chronic nephropathy and glycerol-triggered acute renal failure syndromes including proteinuria, hypoalbuminemia, hyperlipidemia, serum electrolyte unbalance, renal oxidative stress, and pathological damages. NNAV reduced kidney levels of TNF-**α** and IL-1**β**, but it increased the levels of I**κ**B-**α** and inhibited NF-**κ**B p65 nuclear localization. These findings suggest that NNAV may be a valuable therapeutic drug for acute and chronic kidney diseases.

## 1. Introduction 

Adriamycin (ADR) nephropathy is a classic experimental model of chronic kidney disease, which is considered to be an experimental analogue of human minimal lesion nephrotic syndrome [1]. Nephrotic syndrome shows symptoms of hypoalbuminemia, hyperlipidemia, persistent proteinuria, and abnormal renal function. Oxidative stress and inflammation play critical roles in the process of kidney diseases [2]. Previous studies have demonstrated that reactive oxygen species (ROS) are the principal mediators in the development of nephrosis caused by ADR [3]. Under oxidative stress, superoxide dismutase (SOD) serves as a defense mechanism by degrading superoxides, and attenuating local inflammatory reaction [4]. Malondialdehyde (MDA) is the product of lipid peroxidation and thromboxane A2 synthesis and is a commonly measured biomarker of oxidative stress [5]. It has been reported that ADR causes elevations of many inflammatory cytokines including tumor necrosis factor-alpha (TNF-*α*) and interleukin-1*β* (IL-1*β*) [6]. The I*κ*B kinases (IKKs) are activated by TNF-*α* and IL-1*β* [7]. Under normal conditions, NF-*κ*B binds to inhibitory I*κ*B proteins typified by I*κ*B-*α* in the cytoplasm. Proteolysis of I*κ*B-*α* causes the translocation of liberated NF-*κ*B to the nucleus to activate target gene transcription including the pro-inflammatory cytokines TNF-*α* and IL-1*β* [8]. 

 Hypertonic glycerol injection is one of the most frequently used models of experimental acute nephropathy (ARF, acute renal failure) [9, 10]. The pathogenesis of ARF induced by glycerol may involve decreased renal blood flow and reactive oxygen metabolites released from muscle damage [11, 12].

Snake venom is consisted of a mixture of proteins and peptides that have a variety of biochemical and pharmacological functions. Among them, CVF depletes compliment C3 and thus inhibits inflammatory and immune responses [13]. Our recent study also revealed that neurotoxin in cobra venom inhibited inflammatory response [14]. Previous studies have shown that snake venoms have beneficial effects on the treatment of various pathophysiological conditions, including Parkinson's diseases [15], ischemia [16], and pain [14]. Peptide neurotoxins from snake venoms were used for the identification and characterization of membrane ion channels and receptors in human neurons [17]. A short form of neurotoxin cobrotoxin from *Naja naja atra* venom (NNAV) reduced I*κ*B degradation and inhibited the activation of NF-*κ*B [18]. 

It is usually assumed that the oral administration of peptides will be biologically ineffective due to enzymatic digestion or chemical degradation in gastrointestinal tract. However, Giorgi et al. [19] reported that a low molecular weight component from Crotalus durissus terrificus venom induced analgesia when given orally. Similarly, oral administration of neurotoxin from king cobra produced the analgesic action [20]. Recently, the absorption of ^125^I-labled neurotoxin from rectum was also confirmed in rabbits [21]. In our observations, an LD50 (102 mg/kg) of NNAV in mice was reliably established, and substantial absorption of 18[F]-labeled cobrotoxin after mouth administration was also observed (unpublished data). It has been reported that NNAV is used in the clinic for the treatment of diabetic nephropathy [22] with positive results. Therefore, we speculated that oral administration of NNAV might produce the protective effects in rat models of nephropathy. The results indicated that NNAV reduced ADR and glycerol-induced oxidative stress, kidney tissue damage, proteinuria, hypoalbuminemia, and hyperlipidemia.

## 2. Materials and Methods

### 2.1. Animals

Male Wistar rats weighing 180–220 g were obtained from the Shanghai SLAC Laboratory Animal Co. Ltd. Rats were housed in a climate-controlled room (temperature 18–22°C; humidity 55 ± 10%; 12 h light/dark cycle with lights on at 7 : 00 AM) with food and water available ad libitum. Animals were allowed to acclimatize to the laboratory conditions for at least one week prior to the experiments which were carried out in the light phase. Body weight was measured once a week during the entire protocol. All procedures were approved by the Soochow University Animal Care and Use Committee and were carried out in accordance with the National Institutes of Health Guide for the Care and Use of Laboratory Animals (the National Research Council, 1996).

### 2.2. Drug Administration

Dried NNAV powder was purchased from the Rainbow Snakefarm (Yu Jiang, Jiangxi Province, China). The venom was certified for having standard biological activities and compositions. NNAV contains neurotoxin 8%–10%, cardiotoxin 35%–40%, nerve growth factor 1%-2%, phospholipase A2 8%, and cobra venom factor (CVF). The biological activities and compositions were stable under −20°C. Cobra venom solution was freshly prepared in sterile 0.9% saline, heated in boiling water as previously reported [23] and was administered to rats by gastric injection daily at the doses of 30, 90, and 270 *μ*g/kg or 20, 40, and 80 *μ*g/kg in a volume of 2 mL/kg 5 days prior to adriamycin (ADR, doxorubicin hydrochloride) or glycerol injection. For control rats, sterile 0.9% saline solution was taken orally in a volume of 2 mL/kg. The doses of NNAV were decided based on the previous studies on analgesia and on adjuvant-induced arthritis and based on the pilot experiment on adriamycin-induced nephropathy. These dose ranges produced analgesic effects, relieved adjuvant-induced arthritis, and reduced proteinuria in ADR nephropathy in a pilot study. ADR was obtained from Shenzhen Main Luck Pharmaceuticals Inc. (Shenzhen, China). Chronic nephropathy was induced by a single injection of ADR (6 mg/kg body weight [24], dissolved in sterile 0.9% saline solution) through the tail vein. Male Wistar rats were dehydrated for 24 h, and acute nephropathy (ARF, acute renal failure) was produced by intramuscular injection of 8 mL/kg 50% v/v glycerol (Sinopharm Chemical Reagent Co., Ltd., China) in sterile saline (0.9% w/v NaCl) [10]. Control rats were injected with sterile 0.9% saline solution only.

### 2.3. Urine Collection

During the ADR chronic nephropathy experiment, the animals were placed in metabolic cages for 24 h every week for urine collection, with free access to water but without food pellets that could fall into the urine collector and bias the measurement of urinary proteins. In the ARF model, rats were placed in metabolic cages for 24 h for urine collection three days after glycerol was injected, and the rats were then sacrificed. The total urinary protein concentration (grams per liter) was measured with the Coomassie brilliant blue protein assay kit (Nanjing Jiancheng Bioengineering Institute, China) and an ultraviolet spectrophotometer (UV-2600, Shimadzu, Tokyo, Japan).

### 2.4. Blood Serum Parameter Measurement

Five weeks after ADR injection and 72 h after glycerol administration, all rats were sacrificed under general anesthesia using i.p. injection of pentobarbital sodium. Abdominal aorta blood was collected in test tubes, and the serum was separated by centrifugation at 3,000 rpm for 15 min. The serum was stored at −70°C and thawed just before use. Serum levels of total protein (TP), albumin (ALB), globulin (GLB), albumin/globulin (ALB/GLB), urea nitrogen (BUN), creatinine (Cr), total cholesterol (CHOL), triglyceride (TG), potassium (K), phosphorus (P), and cystatin C (Cys-C) were determined with commercially available kits and an automatic biochemistry analyzer (Mindray BS-800, Shenzhen, China). SOD activity in the serum was measured using a colorimetric enzyme assay kit (Beyotime Institute of Biotechnology, China) with WST-1(2-(4-Iodophenyl)-3-(4-nitrophenyl)-5-(2,4-disulfophenyl)-2H-tetrazolium, monosodium salt), a highly water-soluble tetrazolium salt. The serum MDA level was determined based on the reaction of MDA with thiobarbituric acid (TBA) using the Lipid Peroxidation MDA Assay Kit (Beyotime Institute of Biotechnology, China). The activity of SOD and levels of MDA were calculated following the manufacturer's instructions.

### 2.5. Kidney Tissue Sample Preparation

Kidneys were removed and weighed immediately after rats were sacrificed for histological examinations. Coronal sections of renal tissue were immersion-fixed in 10% PBS-buffered formalin and embedded in paraffin. Histological sections were stained with hematoxylin and eosin (HE), periodic acid-Schiff (PAS), and Masson's trichrome. Each section was evaluated with an Olympus light microscopy (Olympus, Tokyo, Japan) with a high-resolution digital camera system. For electron microscopic examination, small blocks of the kidneys were fixed in 4% glutaraldehyde, postfixed in 2% osmium tetroxide, dehydrated in graded ethanol, and then embedded in epoxy resin. Ultrathin sections were stained with lead citrate and uranyl acetate and examined using an electron microscopy (JEM-1230, JEOL, Japan). The remaining cortex of the kidney was stored at −80°C and thawed just before use. 

 The renal cortex was grinded in 2 mL of 50 mmol/L Tris HCl buffer containing 0.1 mmol/L ethylenediaminetetraacetic acid (EDTA) at pH 7.4, homogenized on ice using a homogenizer. After centrifugation at 12000 rpm, 4°C for 5 minutes, the supernatant was collected, and total protein concentration was measured using a BCA kit (Pierce Biotechnology, Waltham, MA, USA). The homogenate was used for the determination of activity of SOD and levels of MDA.

### 2.6. ELISA Assay for Cytokines

The renal cortex was grinded in PBS (pH 7.4) buffer solution and homogenized on ice using a homogenizer. The levels of TNF-*α* and IL-1*β* were determined using the commercially available ELISA kits (Shanghai Hushang Biotechnology Co., Ltd., China). All ELISA assays were performed following the manufacturer's instructions.

### 2.7. Western Blot Analysis

The renal cortex was homogenized using a homogenizer in tissue lysis solution supplemented with protease inhibitors (Protease Inhibitor Cocktail Tablets, Roche, Mannheim, Germany) and phosphatase inhibitors (Phosphatase Inhibitor Cocktail Tablets, Roche, Mannheim, Germany). The lysates were centrifuged at 12,000 g for 15 min at 4°C. Total protein concentration was measured using a BCA kit (Pierce Biotechnology, Waltham, MA, USA). Equivalent amount of proteins was electrophoretically separated on SDS-polyacrylamide gels and transferred to nitrocellulose membranes, which were then blocked with Tris-buffered saline (TBS) containing 5% (w/v) dry milk and 0.1% Tween 20 for 1 h at room temperature. Then the membranes were incubated overnight at 4°C with primary antibody (rabbit monoclonal anti-P-IKK*α* antibody (Cell Signaling Technology, Danvers, MA, USA) or rabbit monoclonal anti-I*κ*B-*α* antibody (Cell Signaling Technology, Danvers, MA, USA)). After washing with TBS containing 0.1% Tween 20, membranes were incubated for 1.5 h at room temperature with fluorescence secondary antibodies (Li-COR Biosciences, Lincoln, NE, USA). The signal was read with ODYSSEY INFRARED IMAGER (Li-COR Biosciences, Lincoln, NE, USA). The signal intensity of primary antibody binding was quantitatively analyzed with Image J Software (W. S. Rasband, Image J, NIH, Bethesda, MD, USA) and was normalized to a loading control *β*-actin (mouse monoclonal antibody, Sigma). 

### 2.8. Immunofluorescence Analysis

Paraffin sections (3 *μ*m) were prepared from embedded kidney tissue mentioned before. Paraffin sections were deparaffinized in xylenes and rehydrated in decreasing grade of ethanol and deionized water, and then they were blocked in PBS containing 1% normal bovine serum albumin and 0.5% Triton X-100 for 1 h at room temperature. Paraffin sections were then incubated with primary mouse monoclonal anti-NF-*κ*B p65 antibody (Cell Signaling Technology) overnight at 4°C. Tissue sections were rinsed three times with PBS and incubated with FITC-conjugated donkey anti-mouse IgG (Jackson ImmunoResearch, Baltimore, MD, USA) for 1 h at room temperature. After being rinsed three times with PBS, the nuclei were counterstained with 4,6-diamidino-2-phenylindole (DAPI), and sections were cover-slipped with fluoromount aqueous mounting medium (Sigma). Images were collected with Nikon C1 laser-scanning confocal unit (Nikon D-Eclipse C1, Tokyo, Japan) adhered to an inverted microscope (Nikon Eclipse TE2000-E).

### 2.9. Statistical Analysis

All data were shown as means ± SD values and analyzed using a one-way ANOVA. Post hoc comparisons were performed using the Student-Newman-Keuls multiple comparison test; *P* < 0.05 was considered statistically significant. Calculations were performed using the SPSS 16.0 statistical package.

## 3. Results

### 3.1. Effects of NNAV on Proteinuria in Acute and Chronic Nephropathy

**Figure 1:**
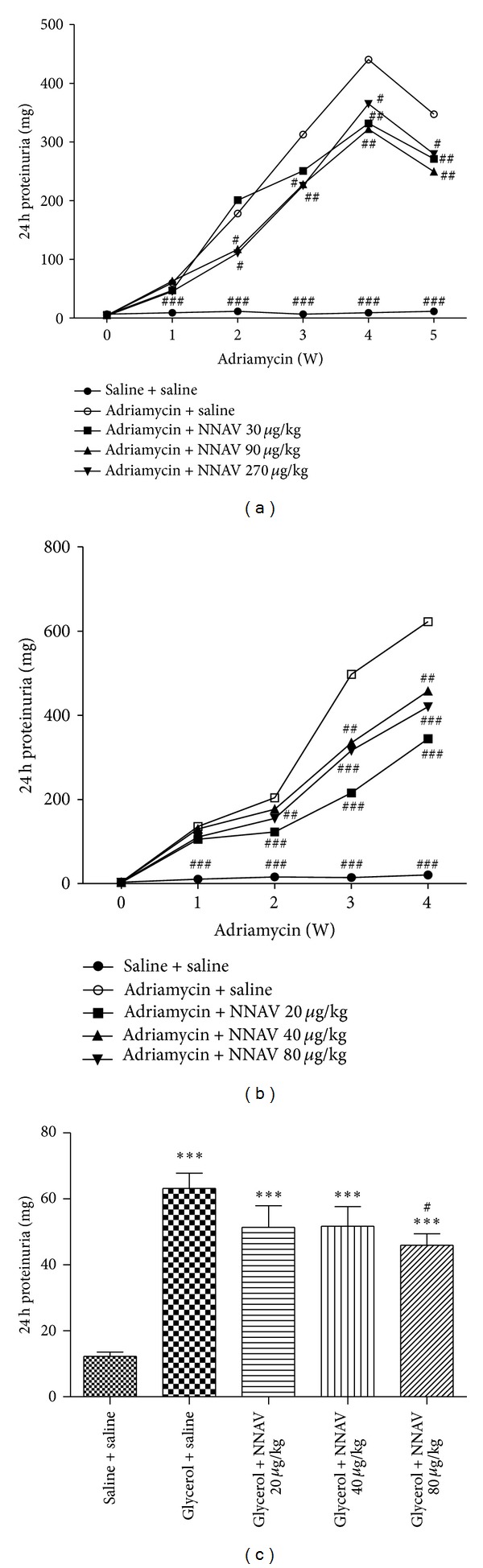
The effects of NNAV on urinary protein excretion. Wistar rats were treated with oral administration of NNAV at a dose of 30, 90, and 270 *μ*g/kg (a) or 20, 40, and 80 *μ*g/kg (b) once a day starting 5 days prior to adriamycin (ADR) injection. ADR (6 mg/kg) was administered by tail vein injection. Wistar rats were treated with oral administration of NNAV (20, 40, and 80 *μ*g/kg) once a day starting 5 days prior to glycerol injection (c). Glycerol (50% v/v, 8 mL/kg) was administered by intramuscular injection. Urine was collected for determination of proteinuria before and after ADR administration and 72 h after glycerol injection. ***P < 0.001 compared with “saline + saline” group; ^#^ *P* < 0.05, ^##^ *P* < 0.01, and ^###^ *P* < 0.001 compared with “adriamycin (or glycerol) + saline” group; *n* = 7–10.

Urine protein excretion is a well-recognized marker of glomerular damage.  Figure 1(a) shows the effects of NNAV on reducing urinary protein excretion after ADR administration. Mean urinary protein excretion was 59.96 ± 22.13 mg/24 h on day 7 and progressively increased to 347.40 ± 46.95 mg/24 h on day 35 after ADR injection in the model rats (adriamycin + saline group). Although urinary protein excretion was also progressively increased with time, the data showed that NNAV significantly reduced output of urinary proteins. The mean urinary protein excretion was 271.00 ± 37.12 (*P* < 0.01, versus model group), 249.40 ± 60.56 (*P* < 0.01), or 279.67 ± 76.27 (*P* < 0.05) mg/24 h on day 35 after ADR in rats treated with NNAV 30, 90, or 270 *μ*g/kg, respectively. In another study, the doses of NNAV were changed to 20, 40, and 80 *μ*g/kg. The results showed that NNAV again markedly decreased ADR-induced proteinuria (Figure 1(b)). As shown in Figure 1(c), the mean urinary protein excretion was 63.18 ± 12.38 mg/24 h at 72 h after glycerol injection in ARF group (glycerol + saline group), while it was 51.41 ± 18.61, 51.79 ± 16.70, and 45.98 ± 9.70 (*P* < 0.05) mg/24 h in rats treated with NNAV 20, 40, or 80 *μ*g/kg, respectively. The results demonstrated that oral administration of NNAV significantly reduced urine proteins in ADR and glycerol-induced chronic and acute kidney diseases.

### 3.2. Effects of NNAV on Body Weight and Kidney Coefficient

**Figure 2:**
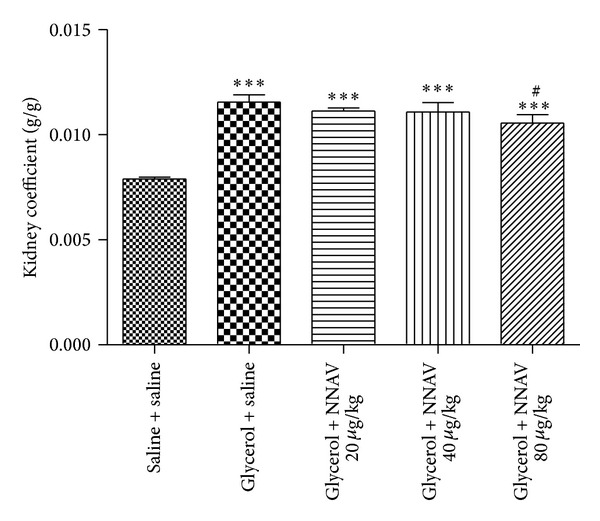
The effects of NNAV on kidney coefficient. Wistar rats were treated as described in the legend of Figure 1. Rats were killed at the end of experiment, and kidneys were dissected for weight measurement. Kidney coefficient was derived by the weight of kidney divided by the total body weight of rat. ***P < 0.001 compared with “saline + saline” group; ^#^ *P* < 0.05 compared with “glycerol + saline” group; *n* = 7–10.

Body weight was measured once a week after ADR injection. Intravenous ADR caused a decrease in body weight [3]. As shown in Table 1, NNAV at the dosages of 90 and 270 *μ*g/kg reduced the weight loss after ADR administration (*P* < 0.001 as compared with model group). Previous study demonstrated that hypertonic glycerol injection would trigger significant increase in kidney weight [25]. The present data (Figure 2) showed that treatment with NNAV decreased the kidney coefficient, especially the group with the dosage of 80 *μ*g/kg (*P* < 0.05 as compared with model group).

### 3.3. Effects of NNAV on Kidney Function

**Figure 3:**
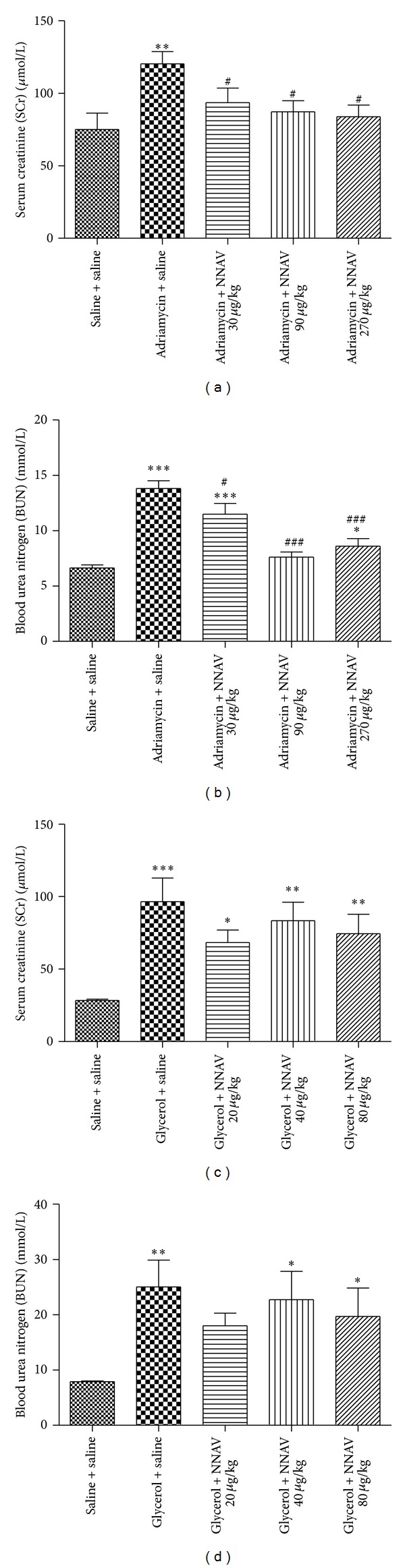
The effects of NNAV on levels of serum creatinine (SCr) and blood urea nitrogen (BUN) in acute and chronic nephropathy rats. Wistar rats were treated as described in the legend of Figure 1. At the end of the experiment, rats were killed and blood samples were collected for determination of serum levels of SCr and BUN in adriamycin ((a) and (b)) and glycerol ((c) and (d)) models. *P < 0.05, **P < 0.01, and ***P < 0.001 compared with “saline + saline” group; ^#^ *P* < 0.05 and ^###^ *P* < 0.001 compared with “adriamycin (or glycerol) + saline” group; *n* = 7–10.

Serum creatinine (SCr) and blood urea nitrogen (BUN) are two principal clusters of renal function variables. Increased levels of SCr and BUN were found in acute and chronic nephropathy (Figure 3). Data demonstrated that the serum levels of SCr and BUN in NNAV-treated groups are significantly lower (*P* < 0.05) than that of ADR-injected model rats (Figures 3(a) and 3(b)). NNAV also decreased the serum levels of SCr and BUN in ARF rats induced with glycerol (Figures 3(c) and 3(d)). 

**Figure 4:**
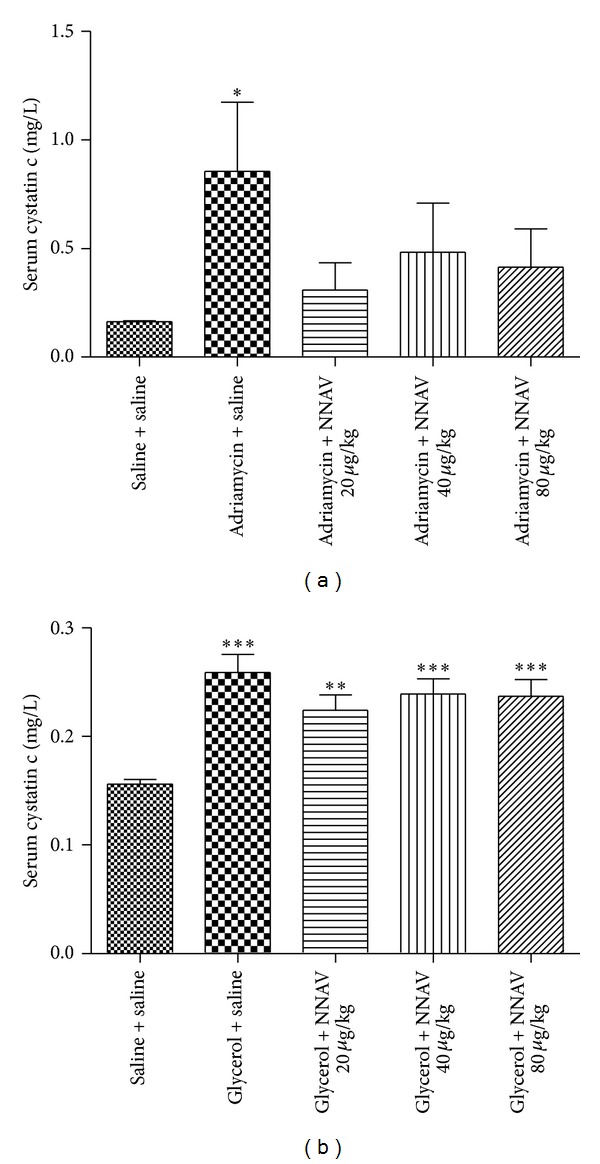
The effects of NNAV on levels of serum Cystatin C in adriamycin and glycerol-injected rats. Wistar rats were treated as described in the legend of Figure 1. At the end of experiment, rats were killed and blood samples were collected for determination of serum levels of cystatin C in adriamycin (a) and glycerol (b) models. *P < 0.05, **P < 0.01, and ***P < 0.001 compared with “saline + saline” group, *n* = 7–10.

The serum level of cystatin C (Cys-C) is a good marker for assessing stable renal functions and their early damage, and it also correlates to direct measures of glomerular filtration rate (GFR) [26]. The serum Cys-C measured in ADR (0.86 ± 0.84 mg/L) and glycerol (0.26 ± 0.05 mg/L) nephropathy models was greatly higher than that in control groups (0.16 ± 0.01 mg/L and 0.15 ± 0.01 mg/L, resp.). NNAV tended to decrease the serum levels of Cys-C, although the effects were statistically insignificant of (Figures 4(a) and 4(b)). All of these results provide evidence that NNAV taken orally can protect the renal function in acute and chronic nephropathy. 

### 3.4. Effects of NNAV on Hyperlipidaemia and Hypoalbuminemia

 Hyperlipidaemia is a risk factor for proteinuria, which promotes glomerular and tubulointerstitial injury and contributes to the progression of kidney disease [27]. The serum total cholesterol (CHOL) and triglyceride (TG) levels in the NNAV-treated group (90 *μ*g/kg) dropped from 13.19 ± 1.41 mmol/L (ADR model) and 26.18 ± 5.37 mmol/L (glycerol model) to 9.37 ± 3.98 and 11.63 ± 9.37 mmol/L, respectively (*P* < 0.05, Table 2). In the present study, the serum levels of albumin in ADR model groups were significantly lower than that of the control group (29.08 ± 4.22 g/L). As shown in Table 2, we found that orally administrated NNAV in a dose of 90 *μ*g/kg (22.26 ± 5.24 g/L) protected the serum albumin from falling (*P* < 0.01) in comparison with the model group (17.16 ± 1.69 g/L). Compared with the model group (47.24 ± 6.09 g/L), the 90 *μ*g/kg NNAV lowered serum GLB levels (35.66 ± 8.32 g/L). Hence, NNAV at a dose of 90 *μ*g/kg elevated the ratio of albumin to globulin in chronic nephropathy rats.

### 3.5. Effects of NNAV on Serum Electrolyte Balance

**Figure 5:**
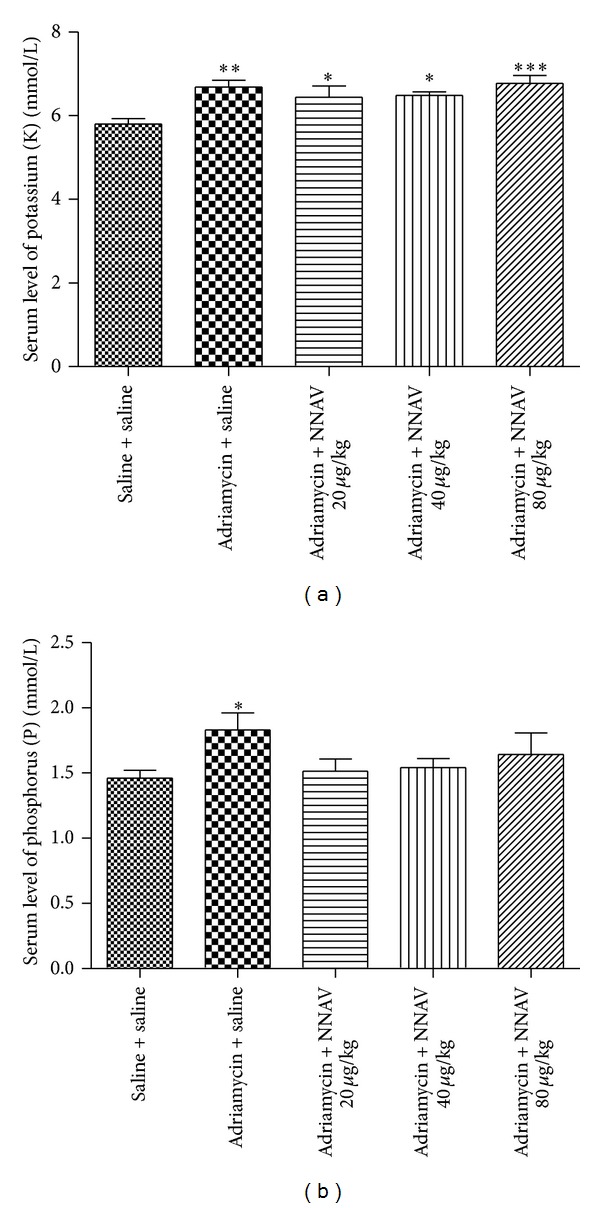
The effects of NNAV on levels of serum potassium and phosphorus in adriamycin-injected rats. Wistar rats were treated as described in the legend of Figure 1. At the end of experiment, rats were killed and blood samples were collected for determination of serum levels of potassium (a) and phosphorus (b). *P < 0.05, **P < 0.01, and ***P < 0.001 compared with “saline + saline” group, *n* = 7–10.

 Under normal conditions, the rats are able to maintain electrolyte balance. Nevertheless, under morbid state, this ability to maintain homeostasis may be lost, making individuals more susceptible to metabolic acidosis [28]. Previous studies showed that there were significant increases in the serum electrolyte concentrations of potassium (K) [29] and phosphorus (P) [30] in ADR-induced chronic nephropathy. The present study found a significant increase in serum levels of potassium (*P* < 0.05); NNAV slightly decreased serum potassium (Figure 5(a)). There were marked increases in the serum levels of phosphorus in the model group; NNAV attenuated elevation of phosphorus (Figure 5(b)).

### 3.6. Effects of NNAV on Oxidative Stress

**Figure 6:**
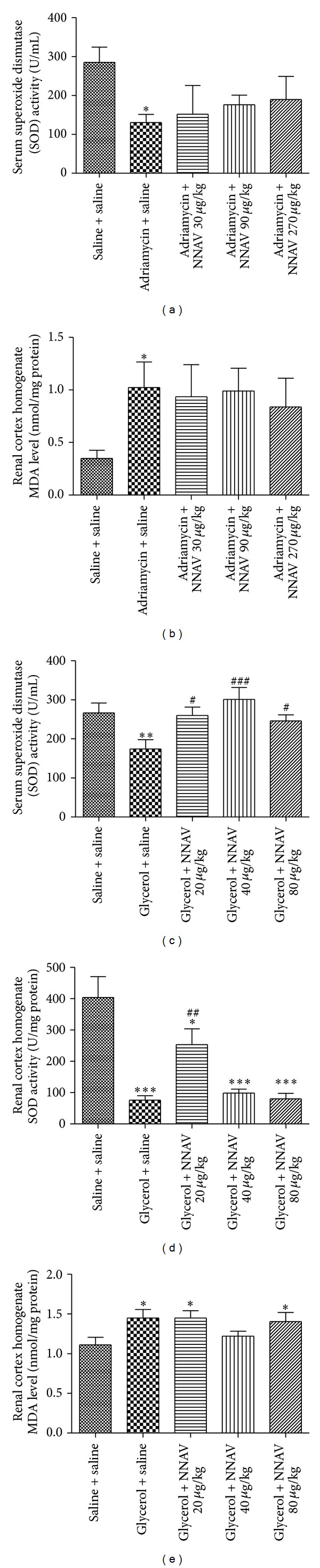
The effects of NNAV on levels of superoxide dismutase (SOD) and malondialdehyde (MDA) in adriamycin and glycerol-injected rats. Wistar rats were treated as described in the legend of Figure 1. At the end of the experiment, rats were killed and blood samples were collected for determination of serum levels of SOD in adriamycin (a) and glycerol (c) models, and renal cortical tissues were dissected for determination of the levels of SOD in adriamycin (b) and glycerol (d) models; meanwhile, the MDA level of renal cortical tissues in glycerol model was measured (d). *P < 0.05, **P < 0.01, and ***P < 0.001 compared with “saline + saline” group; ^#^ *P* < 0.05, ^##^ *P* < 0.01, and ^###^ *P* < 0.001 compared with “adriamycin (or glycerol) + saline” group; *n* = 7–10.

It was demonstrated that SOD activity was lower in acute and chronic nephropathy rats than that in controls; meanwhile, a great increase in MDA level was found in all nephropathy rats. NNAV (30, 90, and 270 *μ*g/kg) increased serum SOD from 130.53 ± 59.71 U/mL in model group to 151.71 ± 165.22 (30 *μ*g/kg), 176.22 ± 65.20 (90 *μ*g/kg), and 189.84 ± 157.18 U/mL (270 *μ*g/kg), respectively (Figure 6(a)). Meanwhile, NNAV decreased renal cortex MDA from 1.02 ± 0.65 nmol/mg protein (ADR model group) to 0.94 ± 0.75 (30 *μ*g/kg), 0.99 ± 0.53 (90 *μ*g/kg), and 0.84 ± 0.67 nmol/mg protein (270 *μ*g/kg), respectively (Figure 6(b)). In an independent study, NNAV increased serum SOD activity from 174.19 ± 74.51 U/mL (ADR model) to 260.29 ± 67.45 (20 *μ*g/kg), 301.15 ± 95.53 (40 *μ*g/kg), and 246.30 ± 45.31 U/mL (80 *μ*g/kg), respectively, and it increased renal cortex SOD from 76.17 ± 40.25 U/mg protein to 253.58 ± 150.30 (20 *μ*g/kg), 99.13 ± 35.66 (40 *μ*g/kg), and 80.66 ± 48.60 U/mg protein (80 *μ*g/kg), respectively (Figures 6(c) and 6(d)). NNAV at a dose of 40 *μ*g/kg decreased the renal cortex MDA from 1.45 ± 0.34 nmol/mg protein (model group) to 1.22 ± 0.20 nmol/mg protein in acute nephropathy rats injected with glycerol (Figure 6(e)).

### 3.7. Effects of NNAV on Renal Pathology

**Figure 7:**
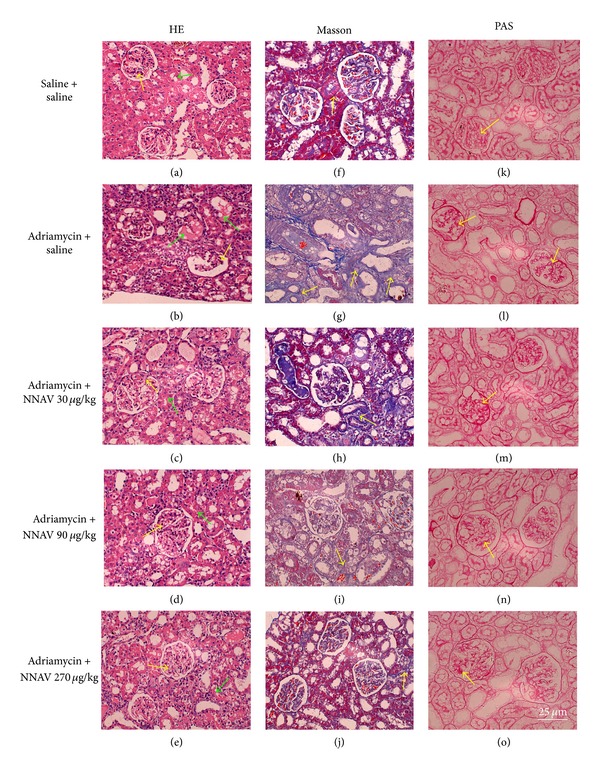
The effects of NNAV on renal pathology in adriamycin-injected rats. Wistar rats were treated as described in the legend of Figure 1. At the end of the experiment, rats were killed and kidneys were dissected and fixed for hematoxylin and eosin ((a)–(e)), Masson's trichrome ((f)–(j)), and periodic acid-Schiff ((k)–(o)) staining. Morphological analysis of renal pathology was performed with a light microscopy. It showed the glomerular deformation and damage (yellow arrows, (a)–(e)), tubular dilatation (green arrows, (a)–(e)), tubule interstitial collagen proliferation (yellow arrows, (f)–(j)), and glomerular basement membrane and mesangial expansion (yellow arrows, (k)–(o)) in the model group. NNAV reduced these pathological changes to a varying degree. Scale bar: 25 *μ*m.

When the renal histological sections were stained with hematoxylin and eosin (HE) and examined with a light microscopy, morphology of glomerular and renal tubule was grossly normal in the control group. Figures 7(a)–7(e) showed that, in the rat kidney of the model group, there were marked pathological lesions characterized by glomerular deformation, stenosis of the renal glomerulus capsular space, and blurry margin between each part. In addition, there were tubular dilatation and abundant protein exudation in the renal tubular lumen and many inflammatory cells around glomerulus. In NNAV-treated groups, these pathological damages were lighter than those of the model group, although glomerular deformation, inflammatory cells around glomerulus, and protein cast in the renal tubular lumen were also sporadically observed. 

Masson's trichrome staining showed pathological lesions characterized by glomerular deformation, wall thickening, portion of adhesions between glomerular capillary plexus and the wall, tubule interstitial collagen proliferation, and many inflammatory cells around the glomerulus in the rat kidney of ADR model group. These pathological damages in the kidney morphology were significantly lighter in NNAV-treated groups than those in the model group (Figures 7(f)–7(j)).

 Periodic acid-Schiff (PAS) stain showed lesions of glomerular sclerosis with glomerular basement membrane and mesangial expansion. NNAV reduced these pathological changes to a varying degree (Figures 7(k)–7(o)).

**Figure 8:**
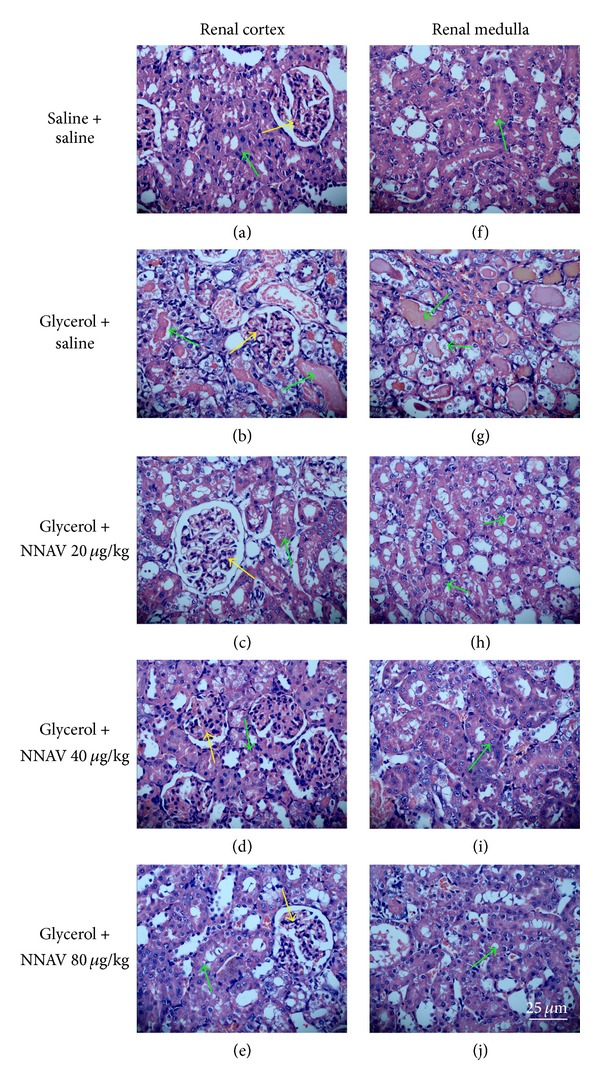
The effects of NNAV on renal pathology in glycerol-injected rats. Wistar rats were treated as described in the legend of Figure 1. At the end of the experiment, rats were killed and kidneys were dissected and fixed for hematoxylin and eosin (HE) staining. Morphological analyses of renal pathology of both cortex ((a)–(e)) and medulla ((f)–(j)) were performed with a light microscopy. It showed the tubular dilatation and necrosis (green arrows) and glomerulus deformation (yellow arrows) in the model group. However, NNAV markedly ameliorated these pathological magnifications. Scale bar: 25 *μ*m.

In glycerol-induced ARF, light microscopy studies showed the following morphological abnormalities: tubular dilatation, intraluminal casts, tubular cell lipid deposition, vacuolization and necrosis, denuded basement membrane, swelling and flattening of tubular cells with brush border loss, tubular interstitial edema, and interstitial inflammatory cell infiltration and deformation of glomerulus due to the compression by dilated tubules. NNAV markedly ameliorated these pathological magnifications (Figure 8). 

### 3.8. Transmission Electron Microscopic Observations

**Figure 9:**
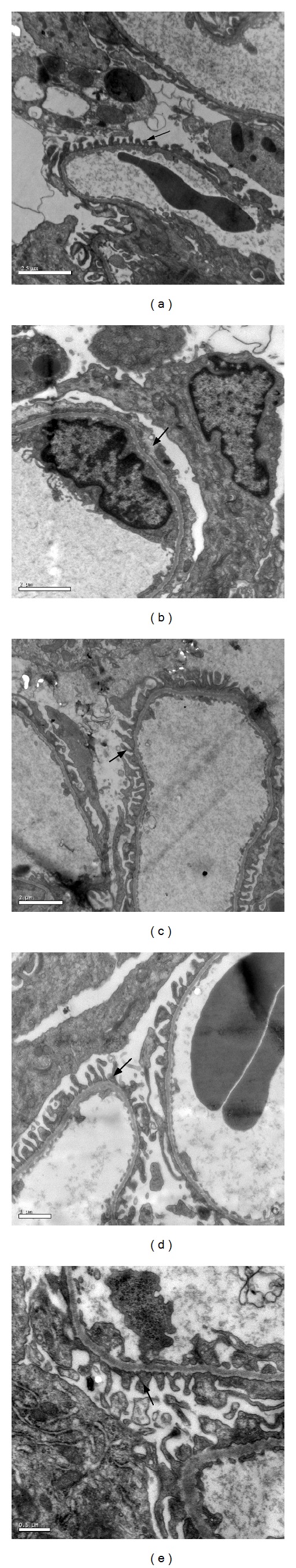
The effects of NNAV on renal pathology in adriamycin-injected rats. Wistar rats were treated as described in the legend of Figure 1. At the end of the experiment, rats were killed and kidneys were dissected and fixed for electron microscopic examination. Morphological analysis of renal pathology was performed with a transmission electron microscopy. (a)–(e) represented control, model, and *Naja naja atra* venom in doses of 20, 40, and 80 *μ*g/kg groups, respectively. It showed foot process effacement (arrows) in model group, but, in *Naja naja atra* venom-treated groups, the foot process lesions (arrows) were significantly reduced. Scale bars: (a) 2.5 *μ*m; (b) 2 *μ*m; (c) 2 *μ*m; (d) 1 *μ*m; (e) 0.5 *μ*m, respectively.

Transmission electron microscopic examination was performed to analyze the ultrastructure changes of rat kidney. In the control group, there were no apparent damages as judged by intactness of renal glomerulus and tubules. Clear foot processes were observed on the visceral surface of renal glomerular epithelial cells. There was no swelling, hypertrophy, and inflammatory cells around them. In the model group, the basal structure of renal glomerulus was damaged, and widespread fusion and effacement of the epithelial foot processes were observed. The line of glomerular basement membrane (GBM) became thickened, wrinkled with unclear margin. In NNAV-treated groups, the kidney lesions were significantly reduced; only minimal fusion and effacement of the epithelial foot processes were observed in the renal glomerulus (Figure 9). 

### 3.9. Effects of NNAV on the Levels of Proinflammatory Cytokines

**Figure 10:**
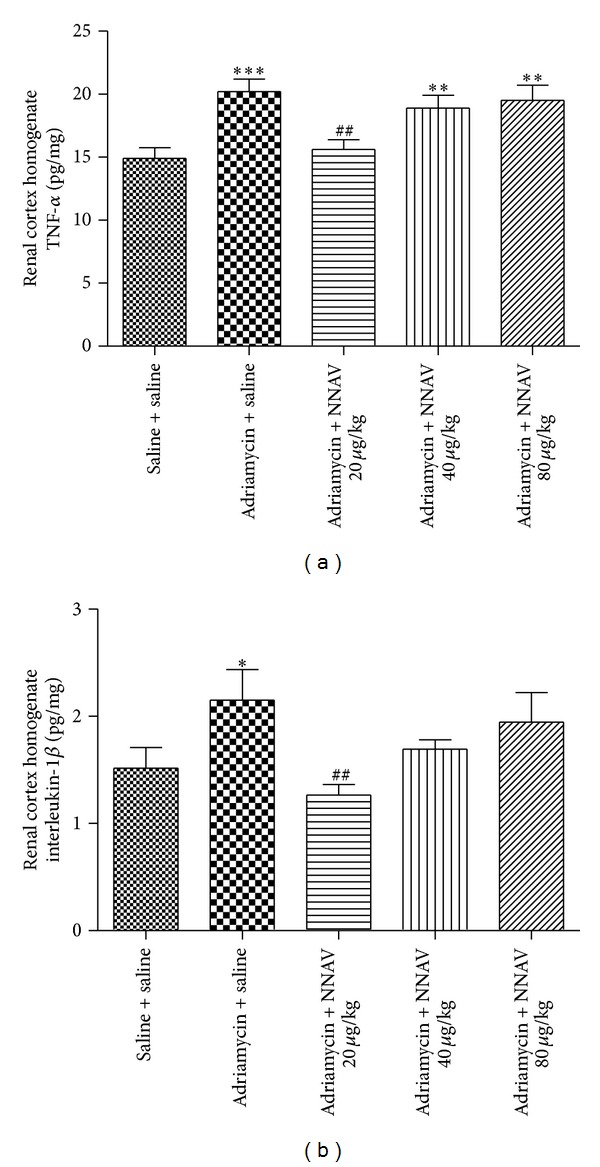
The effects of NNAV on the levels of proinflammatory cytokines. Wistar rats were treated as described in the legend of Figure 1. At the end of the experiment, rats were killed, and the levels of TNF-*α* (a) and IL-1*β* (b) in kidney tissue homogenates were determined using ELISA kits. *P < 0.05, **P < 0.01, and ***P < 0.001 compared with “saline + saline” group; ^##^ *P* < 0.01 compared with “adriamycin + saline” group; *n* = 7–10.

As shown in Figure 10, the levels of TNF-*α* and IL-1*β* in kidney tissue were increased after ADR administration as compared with normal control group. NNAV (20, 40, and 80 *μ*g/kg) decreased the levels of renal cortex TNF-*α* from 20.20 ± 2.65 pg/mg in model group to 15.61 ± 2.15 (20 *μ*g/kg), 18.91 ± 2.80 (40 *μ*g/kg), and 19.51 ± 3.44 pg/mg (80 *μ*g/kg), respectively. Meanwhile, NNAV decreased the levels of IL-1*β* from 2.15 ± 0.75 pg/mg (ADR model group) to 1.27 ± 0.28 (20 *μ*g/kg), 1.69 ± 0.24 (40 *μ*g/kg), and 1.95 ± 0.78 pg/mg (80 *μ*g/kg), respectively (Figure 10).

### 3.10. Effects of NNAV on NF-*κ*B Activation

**Figure 11:**
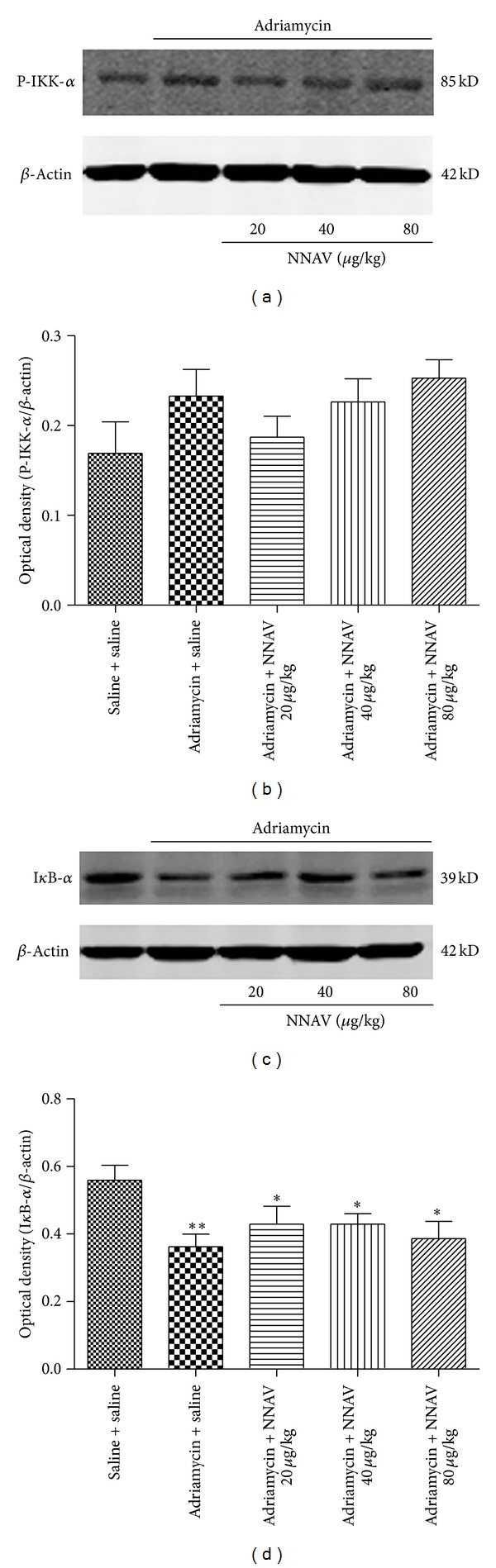
The effects of NNAV on the levels of P-IKK-*α* and I*κ*B-*α*. Wistar rats were treated as described in the legend of Figure 1. At the end of experiment, rats were killed, and levels of P-IKK-*α* (a) and I*κ*B-*α* (c) in kidney tissue homogenates were determined using with Western blot analyses (*n* = 6 experiments). Quantitative analysis of P-IKK-*α* (b) and I*κ*B-*α* (d) were performed with Image J software and normalized to the protein levels of *β*-actin. *P < 0.05 and **P < 0.01 compared with “saline + saline” group.

**Figure 12:**
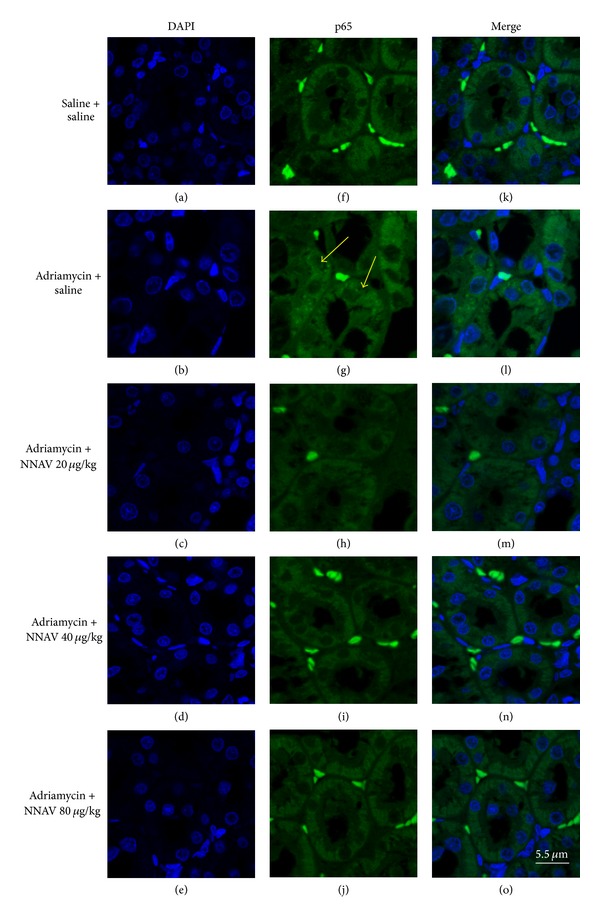
The effects of NNAV on NF-*κ*B activation. Wistar rats were treated as described in the legend of Figure 1. At the end of the experiment, rats were killed, and the nuclear translocation of NF-*κ*B p65 was determined with immunofluorescence with kidney paraffin sections. Nuclei were stained with DAPI ((a)–(e)), and NF-*κ*B p65 was stained with FITC-conjugated donkey anti-mouse IgG ((f)–(j)). Overlay of the images (p65 with DAPI, (k)–(o)) indicated the nuclear translocation of NF-*κ*B p65 (yellow arrows) in some tubular cells. Scale bar: 5.5 *μ*m.

 Activation of the I*κ*B kinase (IKK) may induce the ubiquitylation and eventual proteasomal degradation of I*κ*B-*α*. As shown in Figure 11, the expression of P-IKK-*α* in kidney tissue was upregulated accompanied by the decrease in I*κ*B-*α* protein levels after ADR administration. NNAV slightly reduced the P-IKK-*α* protein levels and significantly recovered the levels of I*κ*B-*α* (Figure 11). Immunofluorescence results showed the upregulation of NF-*κ*B p65 expression in cytoplasm and NF-*κ*B p65 translocation to the nucleus in some tubular cells in ADR model group. NNAV attenuated the NF-*κ*B p65 expression in the cytoplasm and the NF-*κ*B p65 nuclear translocation (Figure 12). 

## 4. Discussion

The present study investigated the protective effects of NNAV on acute and chronic nephropathy in rats. ADR chronic nephropathy is characterized by persistent proteinuria, hypoalbuminemia, hyperlipidemia, edema, hypertension, and abnormal renal function [31]. Hypertonic glycerol injection is a commonly used experimental acute nephropathy (ARF) animal model, and it shows symptoms of rapid decline in GFR, acute tubular cell injury and severe renal dysfunction over hours to days [10]. The current findings demonstrated that the NNAV administrated orally offered the robust protective effects in acute and chronic nephropathy as evidenced by reduced proteinuria, hypoalbuminemia, kidney glomerular and tubular damage, and improved renal functions.

 In our present study, the NNAV significantly reduced ADR-induced weight loss. Previous study showed that hypertonic glycerol injection increased kidney weight [25]. Our present data showed that NNAV decreased the kidney coefficient, especially the group in the dosage of 80 *μ*g/kg. Proteinuria is an important index for the progression and prognosis of renal diseases [32]. The pathophysiological mechanisms underlying proteinuria development may be related to the structure damaged of podocyte foot processes, which makes an important contribution to the molecular sieve for glomerular filtration [33]. Tubular chemokine expression and complement activation that lead to inflammatory cell infiltration in the interstitium may also induce urinary protein excretion [34]. ADR nephropathy demonstrated the feature of selective injury to glomerular podocytes and visceral epithelial cells that maintain the kidney filtration barrier [1]. Podocyte foot processes became thin and flat, widespread fusion and effacement [31]. Tubular atrophy and interstitial inflammatory cells infiltration were also observed in ADR-treated rats [35]. NNAV significantly reduced these pathological changes. With electron microscopy examination, only minimal fusion and effacement of the epithelial foot processes were observed in the renal glomerulus in the NNAV-treated groups, while these were apparent in ADR model rats. 

 GFR is an important marker for renal disease, accepted as the best overall indicator of renal function [36]. Serum SCr and BUN are widely used indices of renal function [37, 38]. The present study demonstrated that serum levels of SCr and BUN were increased in acute and chronic nephropathy rats. NNAV lowered the levels of SCr and BUN compared with the model group. In a recent review, serum level of Cys-C appears to be a promising index for estimating the glomerular filtration rate (GFR) [39], because its serum concentration is independent of muscle mass and does not seem to be affected by age or nutritional status [40, 41]. In the present study, NNAV tended to decrease the elevated serum levels of Cys-C. These results provide further evidence that NNAV can protect the renal function in acute and chronic nephropathy. 

Previous studies have shown that hyperlipidemia aggravates the progression of renal diseases [42, 43]. Renal deposition of lipids is associated with increased proteinuria and glomerulosclerosis in kidney diseases [44]. The relationship between hyperlipidaemia and nephropathy has led to an interest in the potential use of lipid-lowering therapeutic drugs to preserve renal function. Our data showed that 90 NNAV (*μ*g/kg) had a significant lipid-lowering effect compared with model group in ADR model rats. Therefore, the NNAV may be the potential therapeutic drug with lipid-lowering effects to protect renal function. Nephrotic syndrome shows symptom of hypoalbuminemia, which is caused by excessive proteinuria. Serum albumin is widely recognized as a biomarker of underlying inflammation and nutritional status in patients with chronic kidney disease [45], but it is also correlated with age, proteinuria, and hemoglobin levels [46]. On the other hand, the GLB reflects the inflammatory levels in many acute and chronic kidney diseases. The recent study indicated that ADR chronic nephropathy rats acquired lower serum albumin levels together with higher serum globulin levels as compared with the control group. The present data demonstrated that oral administration of NNAV at a dose of 90 *μ*g/kg protected the serum albumin from descending and GLB from elevation, thus elevating the ratio of albumin to globulin.

 Previous research reported that serum electrolytes can be the indices of renal function, in association with changes in urinary composition [47]. Sodium and potassium play an important role in maintaining cell excitability [48]. Under normal conditions, the human beings and rats are able to maintain electrolyte balance. Electrolytes imbalance is present when there is a decrease in GFR with nephropathy. Potassium and phosphate levels increase because of lower GFR [48]. Hyperphosphatemia has been shown to be an independent risk factor for mortality in chronic nephropathy [49]. The results of this study showed a small reduction in serum potassium and phosphorus levels by NNAV. 

 Oxidative stress and inflammation promote kidney injury [50]. Under pathological conditions, oxidative stress is produced by overproduction of ROS or inefficient antioxidant defenses, which appear to be involved in the progression of renal diseases, resulting in kidney damage by lipid peroxidation, DNA damage, and protein modification [2, 51]. SOD is one of the important enzymatic antioxidants, serving as the first line of defense which catalyzes the dismutation of superoxide ions into oxygen and hydrogen peroxide. SOD has an effect on modifying systemic inflammatory responses and antioxidant status [52]. MDA is a product of polyunsaturated fatty acid peroxidation, and plasma malondialdehyde was studied as a marker of oxidative stress and tissue injury [53]. Lower SOD activity and increased MDA level were found in all nephropathy rats as compared with the normal controls in our study. However, NNAV reduced MDA accumulation and increased the activity of SOD. These results suggest that NNAV can reduce oxidative stress. 

 Our data showed that NNAV decreased the levels of TNF-*α* and IL-1*β* in renal cortex as compared with ADR model group. NF-*κ*B is a transcription factor which regulates the expression of several genes encoding proinflammatory mediators, including TNF-*α* and IL-1*β* [54]. NF-*κ*B activation induced nuclear translocation of p65 through proteolysis of the I*κ*Bs [55]. The I*κ*B kinases (IKKs) are activated by various inflammatory factors including TNF-*α* and IL-1*β* [7]. When the IKKs are activated, the I*κ*B-*α* is degraded by the proteasomes. The present study demonstrated that the P-IKK-*α* protein was increased in kidney tissue, while I*κ*B-*α* protein was decreased after ADR administration. Meanwhile, immunofluorescence results showed the increased nuclear localization of NF-*κ*B p65 in ADR model group. These data suggest the activation of NF-*κ*B. NNAV reduced the P-IKK-*α* protein levels and recovered the levels of I*κ*B-*α*, blocked the nuclear translocation of NF-*κ*B p65, and reduced the levels of TNF-*α* and IL-1*β*, suggesting that NNAV may inhibit NF-*κ*B-mediated inflammatory response in kidney. 

It should be noted that there was no linear relationship between doses of NNAV and the pharmacological effects in nephropathy. This type of dose-effect relationship is quite common in the study of Traditional Chinese Medicine due to multiple active components. NNAV contains many peptides with various biological activities. These actions may synergize each other or antagonize each other. Each active component has a different optimal concentration for certain biological actions and has a different rate of metabolism. These may be the reasons why we did not see a linear dose-effect relationship of NNAV.

## 5. Conclusion

 We demonstrated that NNAV can relieve ADR-induced chronic nephropathy and glycerol-triggered ARF by reducing proteinuria, hypoalbuminemia, hyperlipidemia, serum electrolyte imbalance, oxidative stress, and renal pathological damages. NNAV attenuated the inflammatory reactions by inhibiting NF-*κ*B activation. NNAV has a protective effect on renal function. These findings suggest a potential use of NNAV for the treatment of acute and chronic nephropathy. However, whether NNAV is effective when administered after kidney injury needs to be confirmed.

## Figures and Tables

**Table 1:** Body weight changes after adriamycin administration.

	Saline + saline	Adriamycin + saline	Adriamycin + NNAV 30 *μ*g/kg	Adriamycin + NNAV 90 *μ*g/kg	Adriamycin + NNAV 270 *μ*g/kg
First week	38.75 ± 4.77^###^	−0.88 ± 4.22	−9.00 ± 1.31	10.25 ± 3.24^###^	9.75 ± 6.36^###^
Second week	90.50 ± 11.87^###^	14.63 ± 13.64	6.25 ± 9.84	43.25 ± 8.51^###^	47.38 ± 10.93^###^
Third week	110.62 ± 13.75^###^	24.88 ± 9.76	17.50 ± 7.84	57.75 ± 11.45^###^	63.13 ± 13.62^###^
Fourth week	132.00 ± 15.48^###^	30.38 ± 10.51	13.88 ± 10.60	56.63 ± 13.05^###^	63.25 ± 16.93^###^
Fifth week	158.88 ± 16.15^###^	37.13 ± 10.72	9.00 ± 7.96	42.75 ± 17.46^###^	55.00 ± 13.18^###^

The data represent means ± standard deviation. ^###^ *P* < 0.001 compared with “adriamycin + saline” group; *n* = 7–10.

**Table 2:** Serum levels of total cholesterol, triglyceride, albumin, globulin, and albumin/globulin in control and adriamycin chronic nephropathy rats.

	Saline + saline	Adriamycin + saline	Adriamycin + NNAV 30 *μ*g/kg	Adriamycin + NNAV 90 *μ*g/kg	Adriamycin + NNAV 270 *μ*g/kg
Total cholesterol (mmol/L)	2.34 ± 2.98^###^	13.19 ± 1.41***	17.79 ± 2.74***	9.37 ± 3.98^#∗∗∗^	13.32 ± 3.02***
Triglyceride (mmol/L)	1.99 ± 0.56^###^	26.18 ± 5.37***	46.27 ± 12.14***	11.63 ± 9.37^##^	27.08 ± 13.76***
Albumin (g/L)	29.08 ± 4.22^###^	17.16 ± 1.69***	14.11 ± 2.30***	22.26 ± 5.24^##∗∗^	18.86 ± 2.82***
Globulin (g/L)	25.92 ± 3.91^##^	47.24 ± 6.09**	75.05 ± 19.61***	35.66 ± 8.32	53.95 ± 16.88***
Albumin/Globulin	1.15 ± 0.23^###^	0.37 ± 0.06***	0.21 ± 0.10***	0.68 ± 0.33^##∗∗∗^	0.40 ± 0.18***

The data represent means ± standard deviation. **P < 0.01 and ***P < 0.001 compared with “saline + saline” group. ^#^ *P* < 0.05, ^##^ *P* < 0.01, and ^###^ *P* < 0.001 compared with “adriamycin + saline” group; *n* = 7–10.
